# Increased Sensitivity to Binge Alcohol-Induced Gut Leakiness and Inflammatory Liver Disease in HIV Transgenic Rats

**DOI:** 10.1371/journal.pone.0140498

**Published:** 2015-10-20

**Authors:** Atrayee Banerjee, Mohamed A. Abdelmegeed, Sehwan Jang, Byoung-Joon Song

**Affiliations:** Laboratory of Membrane Biochemistry and Biophysics, National Institute on Alcohol Abuse and Alcoholism, Bethesda, Maryland, United States of America; University of Louisville School of Medicine, UNITED STATES

## Abstract

The mechanisms of alcohol-mediated advanced liver injury in HIV-infected individuals are poorly understood. Thus, this study was aimed to investigate the effect of binge alcohol on the inflammatory liver disease in HIV transgenic rats as a model for simulating human conditions. Female wild-type (WT) or HIV transgenic rats were treated with three consecutive doses of binge ethanol (EtOH) (3.5 g/kg/dose oral gavages at 12-h intervals) or dextrose (Control). Blood and liver tissues were collected at 1 or 6-h following the last dose of ethanol or dextrose for the measurements of serum endotoxin and liver pathology, respectively. Compared to the WT, the HIV rats showed increased sensitivity to alcohol-mediated gut leakiness, hepatic steatosis and inflammation, as evidenced with the significantly elevated levels of serum endotoxin, hepatic triglycerides, histological fat accumulation and F4/80 staining. Real-time PCR analysis revealed that hepatic levels of toll-like receptor-4 (TLR4), leptin and the downstream target monocyte chemoattractant protein-1 (MCP-1) were significantly up-regulated in the HIV-EtOH rats, compared to all other groups. Subsequent experiments with primary cultured cells showed that both hepatocytes and hepatic Kupffer cells were the sources of the elevated MCP-1 in HIV-EtOH rats. Further, TLR4 and MCP-1 were found to be upregulated by leptin. Collectively, these results show that HIV rats, similar to HIV-infected people being treated with the highly active anti-retroviral therapy (HAART), are more susceptible to binge alcohol-induced gut leakiness and inflammatory liver disease than the corresponding WT, possibly due to additive or synergistic interaction between binge alcohol exposure and HIV infection. Based on these results, HIV transgenic rats can be used as a surrogate model to study the molecular mechanisms of many disease states caused by heavy alcohol intake in HIV-infected people on HAART.

## Introduction

Currently, approximately 1.1 million people in the United States are living with HIV infection. A recent epidemiological study indicates that one fifth of the HIV-infected people do not know whether they are infected with HIV [1] and continue normal life style of drinking, smoking, and eating western high fat diet. Alcohol abuse is a major problem in HIV-infected population [2] with approximately 50% of these HIV infected people indulging in regular or binge drinking [3–5]. The rate of alcohol abuse among HIV-infected people under medical care has been found to be almost twice greater than that of the general population [6]. Hazardous or binge drinking is known to damage many tissues including the liver, as reported in both experimental models and people. Further, alcohol abuse and/or dependence have been reported to significantly increase the risk of advanced liver diseases in the HIV infected patients [7,8]. In fact, hepatic disease including cirrhosis and carcinogenesis is one of the main causes of death in HIV-infected people [9].

Alcoholic fatty liver disease (AFLD) is a condition characterized by simple hepatic fat accumulation (steatosis) which can advance to more severe liver disease such as steatohepatitis (fatty liver and inflammation), fibrosis, cirrhosis, hepatocellular carcinoma and ultimately liver failure, especially in people who drink more than 60 g/day [10,11]. Inflammation plays a key role in the progression of AFLD as well as in HIV patients since activation of the inflammatory cascade with increased pro-inflammatory cytokine production represents a hallmark of both alcoholic steatohepatitis (ASH), and HIV infection [12,13]. For instance, the levels of monocyte chemotactic protein-1 (MCP-1) and chemokine (C-C motif) ligand 2 (CCL-2) have been reported to be elevated in liver macrophages/Kupffer cells in rodent models of ASH, and these chemokines have been known to promote hepatic steatohepatitis or early inflammatory liver injury [14–17]. Recent results have shown that MCP-1 plays a role in bromodicholoromethane induced infection in obese animals [18]. Further, MCP-1 was considered important in stimulating alcoholic liver injury especially inflammation and steatosis after chronic alcohol feeding whereas MCP-1 knock-out mice exhibited significantly lower hepatic injury than the WT counterparts [17]. In addition, a recent study suggested that even one dose of binge alcohol in humans increased serum endotoxin levels and various cytokines and chemokines including MCP-1, implying the role of gut leakage and MCP-1 in mediating deleterious hepatic effects in these people [19,20]. In addition to MCP-1, leptin, a proinflammatory adipokine, has been known to play an active role in the progression of liver diseases [21–23]. Elevated leptin levels have been observed in patients with non-alcoholic fatty liver disease [24,25]. Moreover, mice deficient in *leptin* gene, have been reported to be resistant to fibrosis [26]. Leptin also enhances the production of pro-inflammatory Th1 cytokines and suppresses the production of anti-inflammatory Th2 cytokines. Chronic alcohol ingestion was also reported to cause myopathy of skeletal muscles in HIV-transgenic (HIV-Tg) rats [27]. Despite many reports on additive/synergistic interactions between alcohol and HIV in extra-hepatic tissues [28–33], the role of HIV in promoting alcohol-induced liver injury has not been studied extensively. Based on the reports described above and many others, it is reasonable to assume that HIV-Tg rats are more vulnerable to alcohol-mediated hepatic injury possibly via increased endotoxin (i.e., gut leakage) and subsequently elevated cytokine/chemokine levels including MCP-1. Therefore, we specifically aimed to evaluate: (1) whether HIV-Tg rats are more susceptible to binge alcohol-mediated gut leakiness, as evidenced by increased serum endotoxin, and inflammatory liver damage than their corresponding WT; and (2) to understand the mechanism of upregulation of endotoxin mediators such as TLR4 and MCP-1, which would promote hepatic inflammation and steatosis in the alcohol-exposed HIV-Tg rats.

## Materials and Methods

### Animal treatment and sample collection

Based on our preliminary results, female rats seem more sensitive to binge-alcohol induced gut leakage than males. Therefore, age-matched 6~8 week old female HIV-Tg or WT Fischer 344 rats, purchased from Harlan Laboratories, were used for the experiments. The HIV-Tg rat strain was genetically prepared to contain the entire genome of the HIV-1 virus except that the 3′ region of the *gag* and the 5′ region of *pol* are deleted [34]. For our experiments, age and gender-matched, HIV-Tg and WT littermates were randomly assigned to four groups and treated with 3 doses of binge ethanol or dextrose in saline (negative control) (n≥4/group): (1) WT-dextrose (WT-control); (2) WT-ethanol (WT-ETOH); (3) HIV-Tg-dextrose (HIV-control); and (4) HIV-Tg-ethanol (HIV-ETOH). Each rat received either 3 consecutive doses of ethanol (3.5 g/dose/kg body weight; oral gavage) or dextrose at 12-h intervals. At 1-h or 6-h following the last dose of ethanol or dextrose, blood and liver tissues were collected for evaluation. These time points were selected based on the several preliminary results to determine the optimal ethanol doses to differentiate the ethanol-mediated effects between HIV-Tg rats and WT. Each experiment was repeated 3–4 times. During the experiment, all the animals were kept in a 12-h light-dark cycle with food and water available *ad libitum*. Following euthanasia, the trunk blood was collected from each rat while the whole liver tissues harvested were divided into two parts. A portion of the largest lobe of each liver was fixed in 10% neutral buffered formalin, while the remaining tissues were snap frozen and stored at -80°C for further analysis. To minimize potential pain and distress, euthanasia was performed by sedating the rats with isoflurane or carbon dioxide inhalation prior to decapitation by guillotine. All animal procedures were carried out in accordance with the NIH guidelines and approved by the Institutional Animal Care and Use Committee of the National Institute on Alcohol Abuse and Alcoholism. For histopathology, 4 μm-thick paraffin-embedded liver sections were cut and stained with H&E for bright-field light microscopy. To assess the degree of inflammation, the number of inflammatory foci per five high power fields (hpf) was quantified from the H&E stained liver sections.

### Isolation and culture of primary hepatocytes and Kupffer cells

Hepatocytes and Kupffer cells were isolated from all mouse groups at 6 h after the last dose of ethanol by *in situ* collagenase perfusion and differential centrifugation on Optiprep (Sigma) density gradient as described previously [35]. Briefly, rat livers were successively perfused *in situ*: first with EGTA solution (5.4 mM KCl, 0.44 mM KH_2_PO4, 140 mM NaCl, 0.34 mM Na_2_HPO4, 0.5 mM EGTA, 25 mM Tricine, pH 7.2), then with perfusion buffer [0.075% collagenase type I in Gey’s balanced salt solution (GBSS) with 0.02% DNase I], and finally with digestion buffer (0.009% collagenase type I in GBSS buffer with 0.02% DNase I) at 37°C for 30–40 min. To isolate the hepatocytes, the liver homogenates were filtered and centrifuged at 25 x *g* for 5 min at room temperature. The pellets obtained (i.e., parenchymal cells—hepatocytes) were briefly washed with PBS and plated on collagen-coated plastic culture dish. To further prepare Kupffer cells, the resulting supernatants were transferred into new tubes and centrifuged at 400 x *g* for 10 min at 4°C. The pellets obtained at this stage were re-suspended in 6 mL of 17% Optiprep, loaded carefully with 3 mL of GBSS washing buffer, and centrifuged at 1,600 x *g* for 17 min at 4°C. The cellular fractions recovered from the interphase between GBSS and 17% Optiprep layers were carefully aspirated, briefly washed with GBSS, and plated on a collagen-coated culture dish. Purity of the cell cultures was assessed by immunohistochemical analyses with the anti-F4/80 antibody. More than 90% of the cells were Kupffer cells confirmed with F4/80 positive staining. Primary hepatocytes and Kupffer cells originally isolated from different rat groups before or after ethanol exposure were used to determine the levels of MCP-1. Hepatocytes were exposed for 1 hr, to neutralizing leptin antibody, before exposing them to various treatment.

### Sample processing

To prepare whole liver homogenates, liver tissues were homogenized in an extraction buffer (50 mM Tris-HCl, pH 7.5, 1 mM EDTA and 1% CHAPS), pre-equilibrated with nitrogen gas to remove the dissolved oxygen, as previously described [36,37]. The concentration of the proteins was determined using the BioRad protein assay kit, following the manufacturer’s protocol as described previously [36,37].

### Determination of serum alanine aminotransferase (ALT) and endotoxin levels

The serum level of ALT of each rat was measured by using the clinical IDEXX Vet Test chemistry analyzer system (IDEXX Laboratories, West Brook, ME, USA). Serum endotoxin levels were determined by using the commercial kit from Lonza (Walkersville, MD) by following the manufacturer’s instructions, as described [38].

### Hepatic triglyceride assay

Liver tissues (50 mg wet weight), homogenized in 5% Triton X-100 solution, were heated in 80–100°C water bath for 2–5 min to solubilize the triglycerides. The samples were then centrifuged at 10,000 x *g* for 10 min, and the collected supernatant was used to determine the triglyceride level by using a kit following the manufacturer’s protocol (BioAssay Systems, Hayward, CA).

### Serum cytokine measurement

Serum MCP-1 level was determined by using the Multiplex bead array assay kit from Millipore (Billerica, MA) and the values were recorded using Luminex xMAP^®^ Technology. Briefly, the levels of the serum cytokines were measured by cytometric quantification method by capturing the target cytokines with spectrally distinct beads pre-coated with a specific capture antibody. Streptavidin-PE conjugate was added to the reaction mixture and incubated to complete the reaction on the surface of each microsphere. The reaction mixture was subsequently directed to pass through a set of laser beam, which excites the dye on the surface of each microsphere. Consequently, the high speed digital signal processor identifies and determines the signal values, based on fluorescent reporter signals.

### Real-time quantitative polymerase chain reaction (Real-time PCR)

TRIZOL® (Life Technologies, Grand Island, NY) was used to isolate total RNA from 50 mg of each frozen liver tissues by following the manufacturer’s protocol. The isolated RNA concentration was measured by Nanodrop® ND-1000 (Thermo Scientific, Wilmington, DE). Real-time PCR amplification reactions (in a final volume of 20 μL) were carried out in an Eco-Real-Time PCR system from Illumina (San Diego, CA) by using Power SYBR® Green RNA-to-CT™ 1-step kit from Life Technologies (Grand Island, NY) according to the manufacturer’s protocols. For the PCR reactions, specific forward and reverse primers (200 nM) and 50 ng for each target RNA were used. A dissociation curve was generated to distinguish the specific amplicons from non-specific amplicons. The Ct-values were calculated with using Eco® software V4.0 (Illumina). The specific primers (shown in Table 1) to analyze the mRNA levels for TLR4, CCR2, leptin, leptin receptor, MCP-1 and beta-actin, respectively, were designed using Primer-BLAST software.

**Table 1:** List of primers used in the study.

Gene name	Primer Sequences (5’-3’)
**TLR4**	F: ATTTACAGAGGGGCAACCGCT
	R: CCAGCCACTGAAGTTGTGAGA
**CCR2**	F: TTCTGGGCTCACTATGCTGC
	R: AAGGGCCACAAGTATGCTGA
**Leptin**	F: AGAAGAAGAAGACCCCAGCGA
	R: CTATCTGCAGCACGTTTTGGGAAG
**Leptin receptor**	F: TGCCTTGGAGGACTATGGGT
	R: AGCCCCCTTCAAAGACGAAG
**MCP-1**	F: CAGCCAGATGCAGTTAATGCC
	R: AGCCGACTCATTGGGATCAT
**Beta-actin**	F: CCCGCGAGTACAACCTTCT
	R: TTCAGGGTCAGGATGCCTCT

### Statistical analysis and others

Data represent means ± SEM from at least three separate measurements, unless otherwise indicated. Sample size was determined using power analysis (http://www.statisticalsolutions.net/pss_calc.php). Statistical analysis was performed using the Student’s *t*-test. Statistical significance was set up at *p*<0.05. The chemicals used in this study were obtained from Sigma Chemical (St. Louis, MO, USA). Other methods or materials, not described in the text, were same as previously published from this laboratory [36, 39–41]. All relevant data are contained in the text and S1 Fig. These data are freely available and can be used by other investigators.

## Results

### Higher hepatic steatosis and inflammation in binge alcohol treated HIV-Tg rats

**Fig 1:**
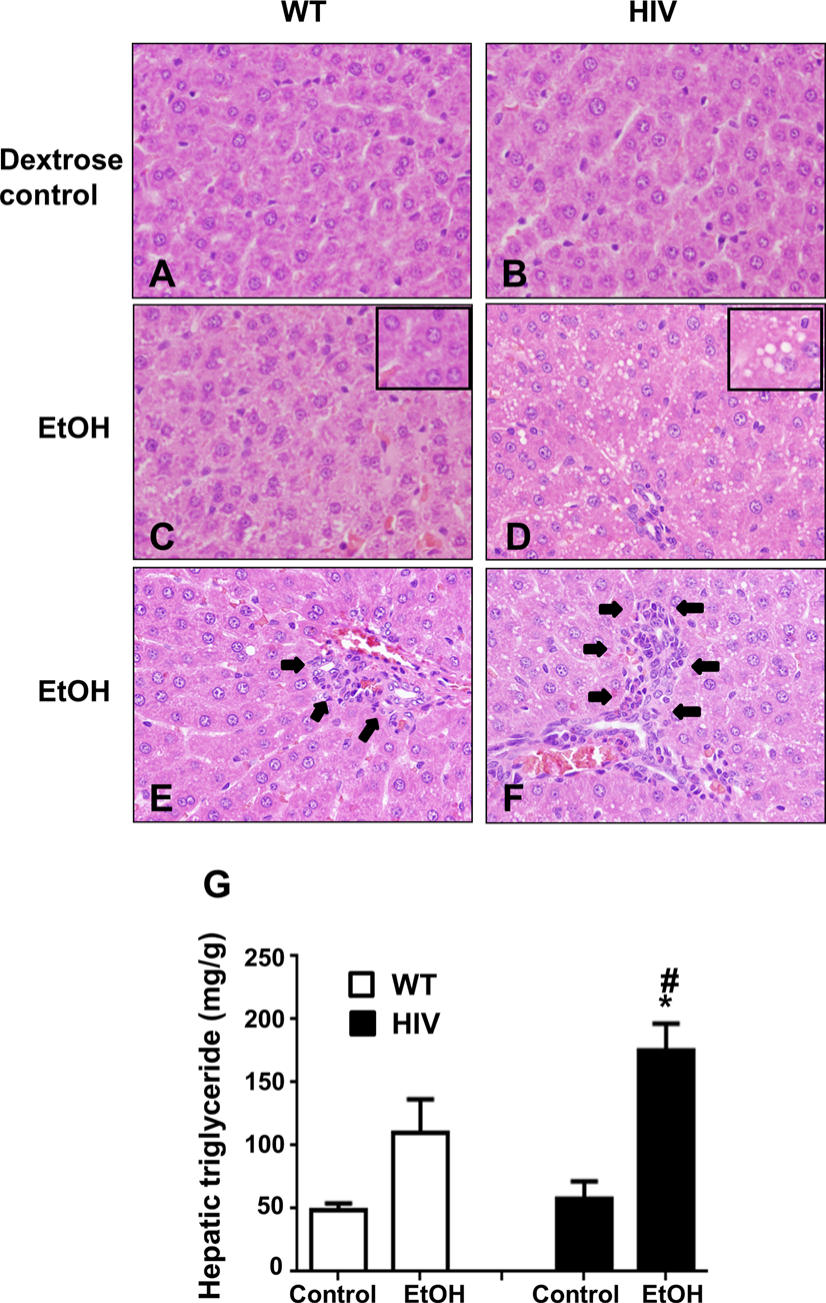
Binge alcohol-mediated hepatic fat accumulation and inflammation in HIV-Tg rats compared to WT rats. (A-F) Representative photomicrographs of H&E stained liver sections from the indicated rat livers are presented: (A) vehicle-control WT, (B) vehicle-control HIV-Tg rats, (C, E) ethanol-exposed WT rats, and (D, F) ethanol-exposed HIV-Tg rats. Insets represent enlarged portions of liver sections to show accumulated fat. Arrows (in E and F) represent accumulated neutrophils. (G) Hepatic triglyceride levels in WT or HIV-Tg rats exposed to dextrose control or ethanol, as indicated. *, # Significantly different from the corresponding dextrose controls and ethanol-exposed WT counterparts, respectively.

Our recent results showed that binge alcohol (3 oral doses at 6 g/kg at 12-h intervals) caused gut leakiness and inflammatory liver injury in WT mice [38]. However, our preliminary results indicated that 3 consecutive oral administration of ethanol at greater than 4 g/kg at 12-h intervals caused gut leakiness in both WT and HIV-Tg rats (data not shown). Therefore, we conducted pilot experiments carefully to determine the optimal ethanol dose for distinct responses between the WT and HIV-Tg rats. Our results clearly showed that 3 doses of ethanol at 3.5 g/kg/dose showed significant gut leakiness and inflammatory liver injury only in HIV-Tg rats. Based on these preliminary results, we used the regimen of 3 consecutive doses (3.5 g/kg/dose) of ethanol at 12-h intervals for all experiments. Histological analysis with H&E staining revealed that the liver of WT Fishers rats and HIV-Tg rats treated with Dextrose (Control) appeared to be normal (Fig 1A and 1B). However, the liver of the HIV-Tg rats exposed to binge alcohol (HIV-EtOH) exhibited significantly higher micro-vesicular fat accumulation (steatosis) and inflammatory foci (Fig 1D and 1F), compared to the corresponding ethanol-exposed WT rats (WT-EtOH, Fig 1C and 1E). The histological appearance of micro-vesicular fat was further supported by the significantly higher levels of hepatic triglycerides (Fig 1G) in the HIV-EtOH rats, compared to those in the other groups. However, serum ALT levels were not significantly different between the groups, although a trend of increased ALT was observed in HIV-EtOH rats compared to other groups (data not shown).

### Binge alcohol treated HIV-Tg rats are more susceptible to ethanol-induced hepatic inflammation

**Fig 2:**
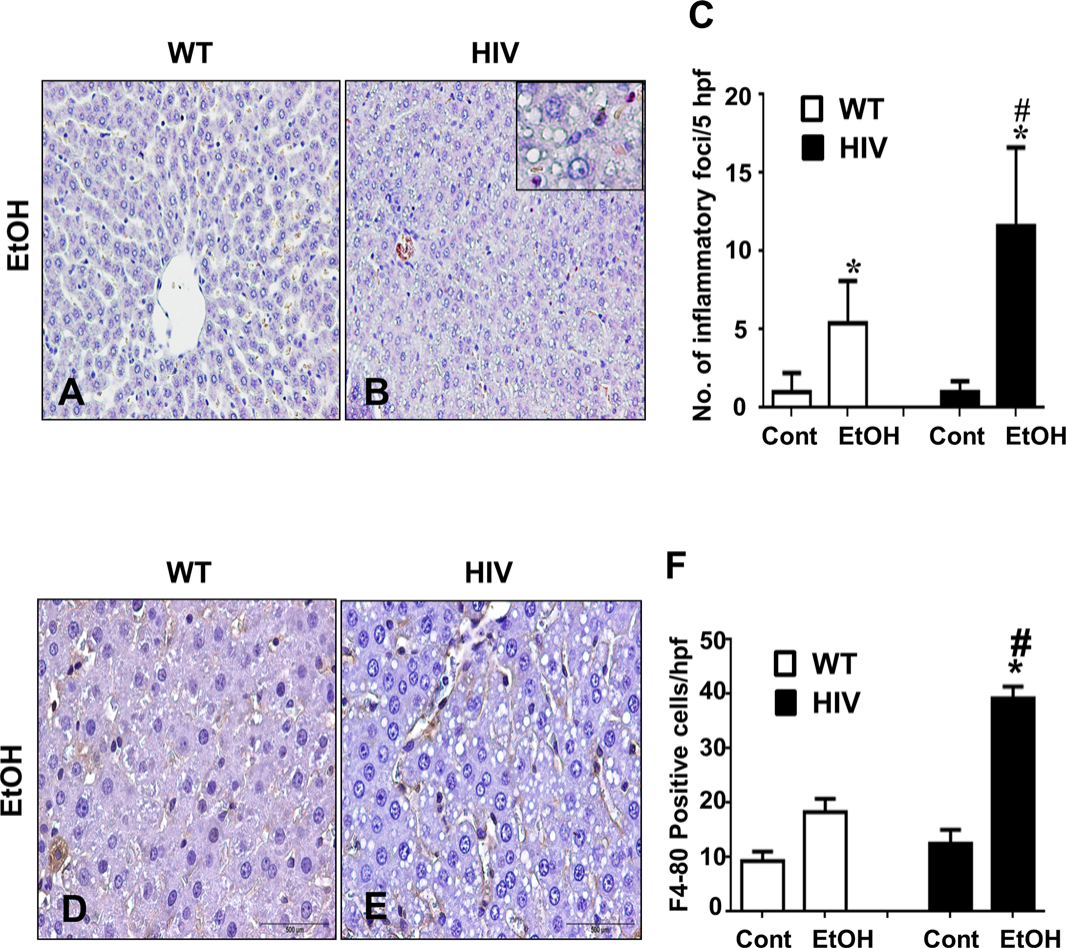
Binge ethanol-mediated increment of inflammatory foci and neutrophils in HIV-Tg rats compared to the corresponding WT rats. (A-C) Representative liver sections stained with chloroacetate esterase indicating inflammatory foci are presented: (A) WT-EtOH, (B) HIV-EtOH, and (C) quantification of inflammatory foci. (D-F) Increase neutrophils stained with F4/80 in liver sections of each group are presented, as indicated: (D) WT-EtOH, (E) HIV-EtOH, and (F) Quantification of F4/80 positive cells per hpf.

Ethanol treatment increased the number of inflammatory foci (Fig 2A and 2B) and neutrophils (chloroacetate esterase staining summarized in Fig 2C) both in the HIV-Tg and WT rats compared to their corresponding controls, but such increase was significantly higher in HIV-EtOH rats, suggesting that these HIV-Tg rats are more susceptible to ethanol-induced inflammation than the corresponding WT. Further, hepatic expression of F4/80 (marker for macrophages) was also examined to determine infiltration of macrophages. Hepatic expression of F4/80 increased significantly in HIV-EtOH group (Fig 2E and 2F), compared to its WT counterpart and control groups (Fig 2D and 2F). Taken together, these results suggest that infiltration of inflammatory cells and hepatic inflammation are significantly elevated in binge-alcohol exposed HIV-Tg rats, compared to the corresponding WT.

### Increased gut leakage in binge alcohol-exposed HIV-Tg rats

**Fig 3:**
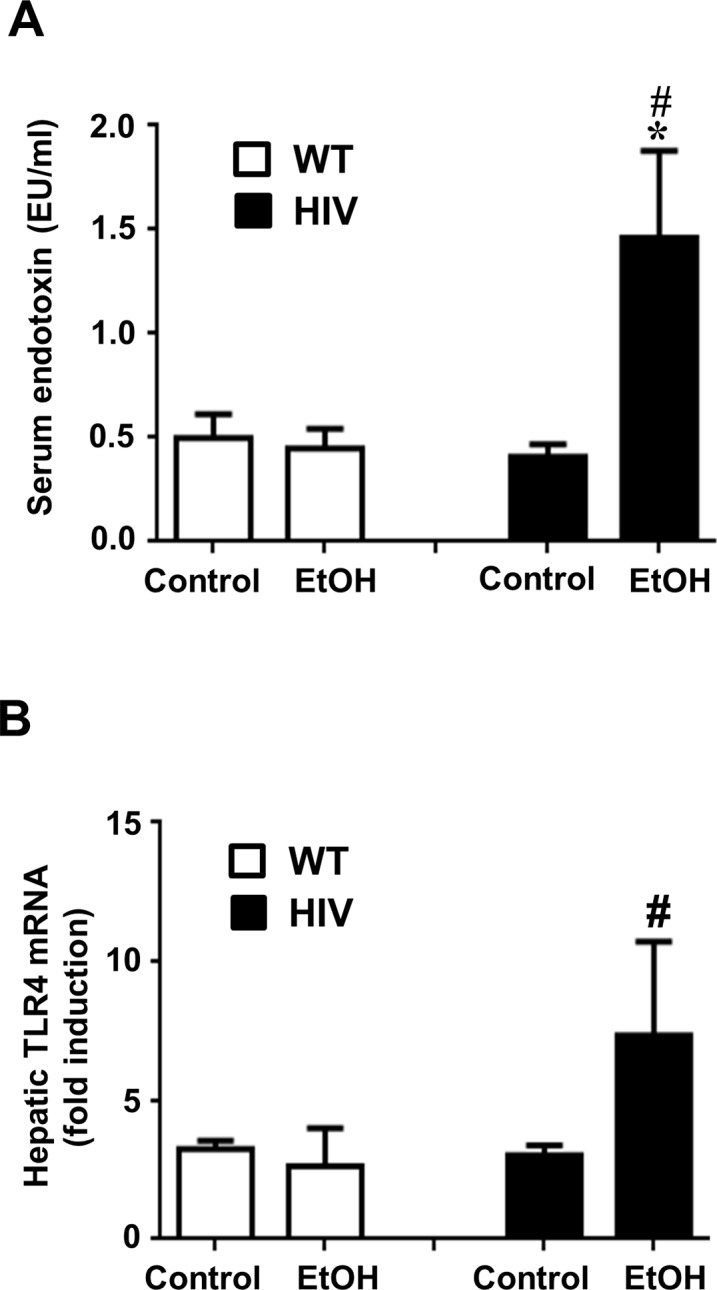
Binge ethanol-mediated elevation of serum endotoxin and TLR4 mRNA levels in HIV-Tg rats compared to WT. Increased gut leakage in ethanol-exposed HIV-Tg rats. (A) Serum endotoxin levels and (B) hepatic levels of TLR4 mRNA in samples collected at 1 and 6 h, respectively, after the last ethanol dose in the indicated groups are presented.

Increased endotoxin/lipopolysaccharide (LPS) due to gut leakage has been known to play a key role in the promotion and progression of inflammatory alcoholic liver disease [42,43]. Significantly higher endotoxin levels were observed in the sera of HIV-EtOH rats, compared to those of the corresponding WT-EtOH rats or their respective controls (Fig 3A). It is well-established that serum endotoxin has been reported to interact with Toll-like receptor 4 (TLR4) in the liver, leading to promoting inflammatory liver injury [44]. Consistent with the results of the serum endotoxin levels, significantly higher expression of TLR4 mRNA was also observed in the liver of HIV-EtOH rats, compared to the corresponding WT and their control rats (Fig 3B). These results indicate that HIV-Tg rats are more susceptible to alcohol-induced gut leakiness than the WT rats and that gut leakiness in the HIV-Tg rats can be induced by this dose of ethanol (i.e., 3.5 g/kg/dose).

### Binge alcohol induces MCP-1 in HIV-Tg rats

**Fig 4:**
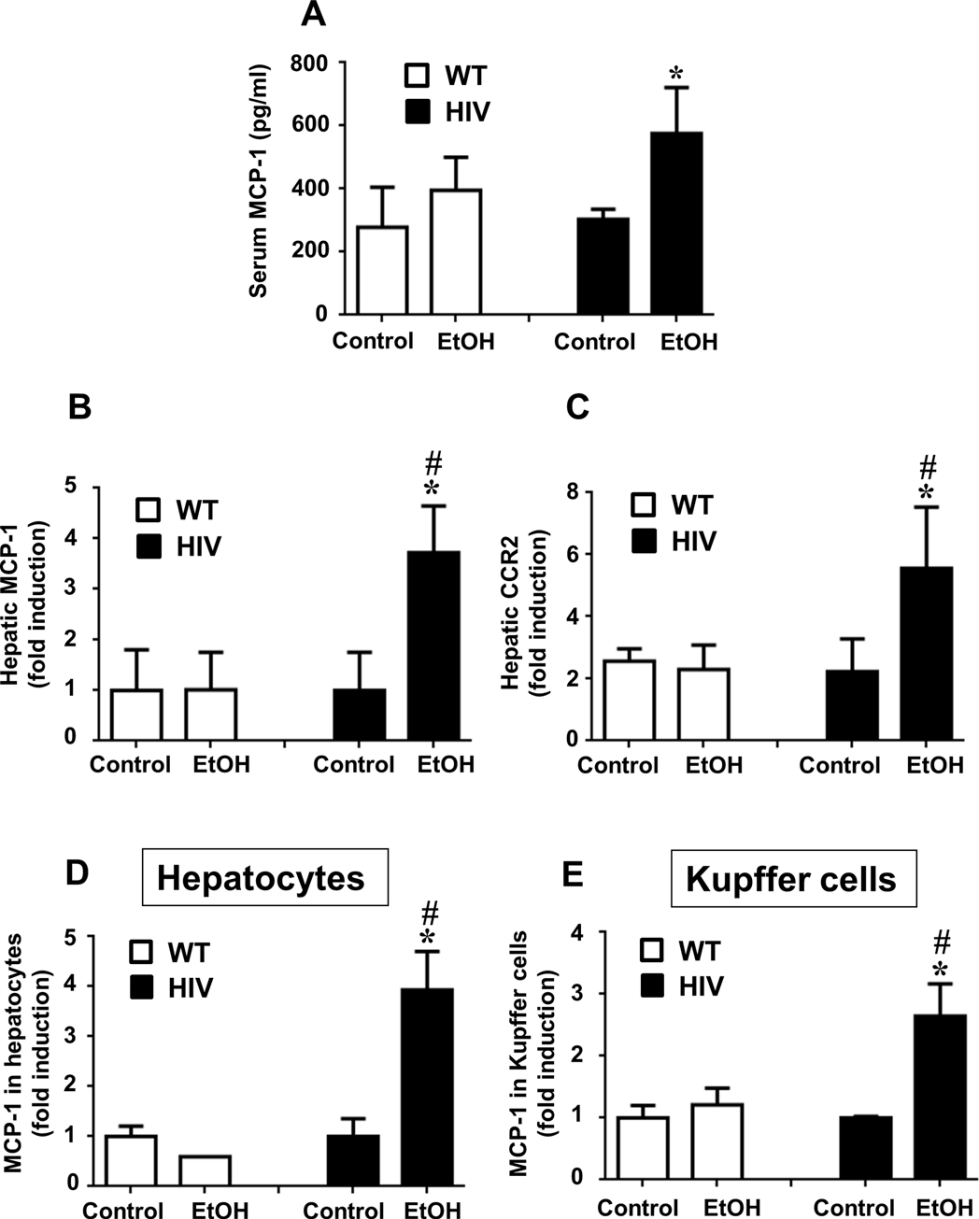
Binge ethanol-mediated elevation of hepatic MCP-1 levels in HIV-Tg rats compared to WT. Changes in MCP-1 levels in HIV-Tg or WT rats following exposure to dextrose control or EtOH are presented. The levels of (A) serum MCP-1, (B) hepatic MCP-1 mRNA, (C) hepatic CCR2 mRNA, (D) MCP-1 mRNA in primary hepatocytes, and (E) MCP-1 mRNA in Kupffer cells in the indicated groups are presented.

Hepatic MCP-1, elevated in chronic ALD, is known to promote neutrophil infiltration into the liver [17]. Therefore we investigated the expression of MCP-1 in control or binge alcohol-exposed HIV-Tg and WT rats. There was no significant difference in the basal levels of MCP-1 between WT and HIV-Tg rats (Fig 4A). Our preliminary results also showed that the plasma levels of major cytokines/chemokines TNF-α, IL-6, MCP-1 and CCR2 were similar between WT and HIV-Tg rats (S1 Fig). However, binge alcohol exposure significantly elevated serum MCP-1 expression in the HIV-EtOH rats, compared to the WT-EtOH rats or its respective controls (Fig 4A). A significant increase in MCP-1 mRNA (Fig 4B) was also observed in the liver of HIV-EtOH rats, compared to the other three groups. MCP-1 has been known to activate inflammatory cells in the liver through its receptor C-C chemokine receptor type 2 (CCR2). Consistent with the elevated MCP-1 levels in HIV-EtOH group (Fig 4B), the expression of hepatic CCR2 mRNA was significantly increased in HIV-EtOH rats, compared to those in the other groups (Fig 4C). To further identify the source of MCP-1 in the liver, we prepared primary culture of hepatocytes and Kupffer cells from all mouse groups, and determined the MCP-1 mRNA levels in these parenchymal and non-parenchymal cell types. Both hepatocytes (Fig 4D) and Kupffer cells (Fig 4E) showed significantly higher levels of MCP-1 mRNA in HIV-ETOH rats, compared with the corresponding WT-EtOH rats and their respective controls. Thus the above results indicate that higher inflammation in binge alcohol-exposed HIV-Tg rats is likely mediated by gut leakiness, TLR4, and MCP-1 in a CCR2 dependent manner and that elevated hepatic MCP-1 in HIV-EtOH rats can be derived from both hepatocytes and Kupffer cells.

### Mechanism of upregulation of TLR4 and MCP-1 in ethanol-exposed HIV-Tg rats

**Fig 5:**
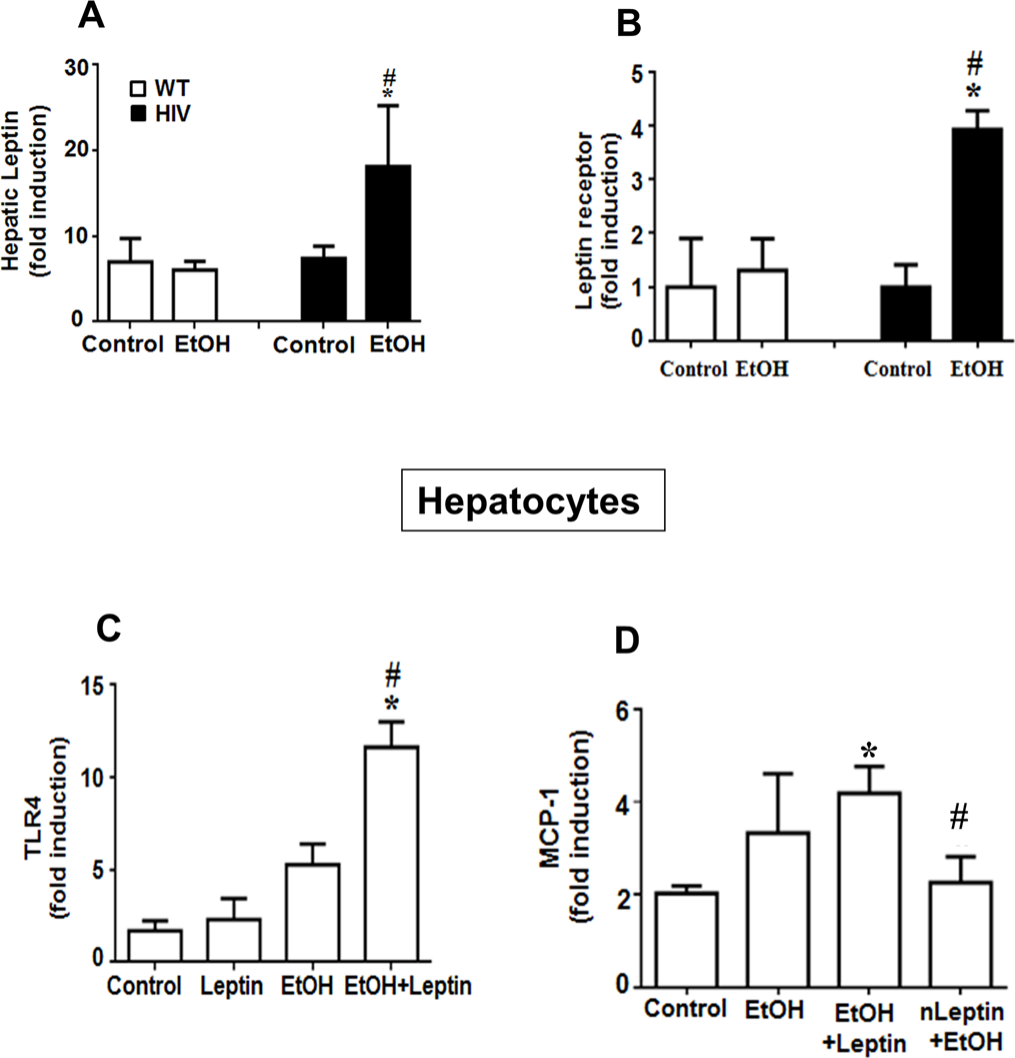
Binge ethanol-mediated elevation of hepatic leptin in HIV-Tg rats compared to WT. Changes in the leptin and its related factors in HIV-Tg or WT rats following exposure to dextrose control or EtOH are presented. The mRNA levels of (A) hepatic leptin, (B) leptin receptor, (C) TRL4 in hepatocytes treated with leptin and EtOH, alone or in combination, (D) MCP-1 in hepatocytes treated with EtOH, leptin, leptin+EtOH or neutralizing anti-leptin (nLeptin) antibody are shown.

The role of leptin was investigated to understand the mechanism of upregulation of TLR4 and MCP-1 in the HIV-EtOH rats in our model. Ethanol-exposed HIV-Tg rats had significantly higher levels of leptin (Fig 5A) and leptin receptor (Fig 5B), as compared to the WT-EtOH rats and their respective controls. Hepatocytes isolated from HIV-control rats were treated with leptin or ethanol alone or in combination. Hepatocytes treated with EtOH+leptin had a significantly higher level of TLR4, as compared to the group treated EtOH or leptin alone and control, indicating that EtOH+leptin upregulates TLR4 mRNA (Fig 5C) in this model. Further, to understand the role of leptin in the upregulation of MCP-1, hepatocytes isolated from HIV-control rats were also treated with EtOH+leptin. Hepatocytes treated with EtOH+leptin had a significantly higher level of MCP-1, compared to the control. Pretreatment with neutralizing anti-leptin antibody (nLeptin) abrogated the effect, indicating the role of leptin in the up-regulation of MCP-1 (Fig 5D).

## Discussion

The incidence of alcohol abuse is remarkably high in HIV-infected people [3–5], leading to increased risk of the development of the progressive stages of ALD including hepatitis, fibrosis/cirrhosis and cancer [9]. However, the underlying mechanism(s) for accelerated liver injury in HIV-infected individuals is poorly understood. Herein, we report for the first time a novel mechanism causing markedly high levels of hepatic steatohepatitis in binge alcohol-exposed HIV-Tg rats, which were used as a model for studying the conditions of HIV-infected people being treated with the highly active anti-retroviral therapy (HAART), since HIV viral replication is suppressed [45]. In this model, binge alcohol caused gut leakiness with elevated levels of endotoxin, leading to up-regulation of hepatic expressions of leptin, TLR4, MCP-1 and CCR2 with increased infiltration of inflammatory cells, all of which likely contribute to inflammatory liver injury in HIV-Tg rats. Based on our results, it is likely that HIV-infected people with or without HAART undergo accelerated progression of inflammatory liver disease when they drink excessive amounts of alcohol.

Epidemiological studies have reported liver disease as the cause of death in 15–17% of HIV-infected people where approximately half of them excessively consume alcohol [4,5]. In a randomized study, Hadigan et al. [46] have identified hepatic steatosis in about 42% of HIV infected subjects, when determined by magnetic resonance spectroscopy. If these individuals continue drinking alcohol, they are more likely to have advanced and accelerated progression of liver disease, as compared to their uninfected counterparts, as recently reported [9]. This trend is in agreement with our animal model, where HIV-Tg rats were more sensitive to the development of alcohol-mediated inflammatory liver disease than compared to the corresponding WT rats or their respective controls when they were exposed to the pre-determined three consecutive ethanol doses (3.5 g/kg/dose at 12-h intervals). However, our preliminary results also showed that gut leakage and inflammatory liver injury could be also observed in WT rats, when they were exposed to ethanol doses greater than 4 g/kg/dose (data not shown). The ethanol dose-related distinct responses in gut leakage and inflammatory liver disease do not appear to be due to different rates of alcohol metabolism between WT and HIV-Tg rats, as reported [47]. Significant increase in inflammation was also supported by the presence of both neutrophils and macrophages in the liver parenchyma, indicating acute alcoholic liver injury in the binge alcohol-exposed HIV-Tg rats, although there was no significant difference in the ALT levels among the four experimental groups despite the increased tendency in HIV-EtOH than the WT-EtOH rats. The lack of significant increase in ALT levels is not uncommon in the presence of hepatic injury and is in agreement with several previous studies, where more severe hepatic inflammatory disorders were observed in the absence of elevated ALT levels [38,48–50].

The important role of gut leakiness and gut derived endotoxin LPS in humans as well as in animal models of alcoholic liver disease are well-established [42,43]. Binge alcohol exposed mice showed increased serum endotoxin levels [38] most likely through the degradation of intestinal tight junction proteins [51]. Previous studies have reported that ethanol exposed HIV-Tg rats are more susceptible to barrier dysfunction, when compared to their control counterparts. For instance, cultured alveolar epithelial monolayer cells prepared from alcohol-fed HIV-Tg rats had increased paracellular permeability to sucrose, compared with monolayer cells from alcohol-fed WT rats and untreated HIV-Tg rats [52]. Alveolar epithelial barrier dysfunction correlated with alterations in the expression of the tight junction proteins occludin and ZO-1. Consistent with these studies, HIV protein induced blood-brain barrier dysfunction has been associated with fragmentation/modification or absence of the tight junction membrane proteins and increased permeability [53,54]. Furthermore, Brenchley et al. [55] have reported that HIV infection is also associated with increased serum endotoxin/LPS levels, suggesting HIV-mediated gut leakiness. HIV infected individuals were also reported to have significantly higher serum endotoxin levels, as compared to the healthy controls [56,57].

TLR4 has been well-established as one of the essential components of the LPS receptor complex necessary for mediating LPS effects [43,58]. Indeed, the role of TLR4-activated chemokines including MCP-1, using TLR4-KO mice, in mediating chronic ethanol-induced neuroinflammation and anxiety-related behavior has been reported [59,60]. Further, in activated human hepatic stellate cells, LPS was found to utilize the components of the TLR4 signal transduction cascade to up-regulate chemokines such as MCP-1 and adhesion molecules, suggesting its role in promoting inflammatory liver injury [61]. In addition, C3H/HeJ mice were known to respond poorly to LPS due to a missense mutation within the coding region of the *Tlr4* gene [62,63], indicating the importance of TLR4 in promoting LPS-mediated hepatic inflammation and toxicities. Consistent with these reports, in our model only the binge alcohol-exposed HIV-Tg rats had significantly higher endotoxin levels and subsequently elevated TLR4 and MCP-1 than those of the other three groups including the WT-EtOH. These results indicate that increased endotoxin through gut leakage is likely to contribute to inflammatory liver disease in alcohol-exposed HIV-Tg rats.

MCP-1 has been established as a master regulator of macrophage function in chronic inflammatory disease. Recent studies have also suggested the important role of MCP-1 in promoting both alcoholic and non-alcoholic liver disease [17,64]. Mice deficient in *MCP-1* gene have been reported to be protective against chronic alcoholic liver injury, as indicated by decreased levels of serum ALT, inflammation and steatosis [17]. Serum MCP-1 concentrations were reportedly significantly increased in patients with alcoholic liver disease and positively correlated with the severity of hepatic inflammation [16]. In addition, patients with severe alcoholic hepatitis and cirrhosis have been reported to have significantly higher MCP-1 levels in their livers [14]. The MCP-1/CCR2 axis in the liver was shown to play a critical role in the migration of inflammatory cells, leading to hepatic fibrosis and cirrhosis [65,66]. Furthermore, pharmacological inhibition of MCP-1 significantly reduced the rates of liver fibrosis by suppressing macrophage infiltration in two different models of fibrosis using carbon tetrachloride and methionine-choline deficient diet [67]. Consistent with these reports, our results showed that binge alcohol-exposed HIV-Tg rats have significantly higher levels of serum and hepatic MCP-1 mRNA, as compared to those of the corresponding WT rats and their respective controls. In parallel, the up-regulated expression of CCR2 mRNA was also observed only in the HIV-EtOH rats, indicating that in our model, MCP-1 was likely to mediate its action via its receptor CCR2. Besides pro-inflammatory response facilitated by MCP-1, recent studies have also suggested the role of this chemokine in promoting hepatic steatosis or early alcoholic liver injury. MCP-1 knock-out mice fed a Lieber-DeCarli alcohol liquid diet for 5 weeks were protected from hepatic steatosis and inflammation, whereas chronic alcohol feeding showed fatty liver with elevated levels of MCP-1 both in the hepatocytes and Kupffer cells in WT mice [17]. These results are in agreement with our current results, where binge alcohol caused steatohepatitis with significantly increased the MCP-1 levels both in the hepatocytes and Kupffer cells in HIV-Tg rats, compared to the corresponding WT rats and their respective controls, further supporting the functional role of MCP-1 in steatosis and inflammatory response. However, additional studies are needed to understand the precise role of MCP-1 in this model.

In addition to TLR4 and MCP-1, leptin has also been known to play an active role in the progression of liver diseases [11–13]. Recent studies have demonstrated the mechanism of leptin-mediated upregulation of CD14 and TLR4 in nonalcoholic steatohepatitis [68]. Endothelial cells treated with leptin have been reported to have greater production of reactive oxygen species accompanied by upregulation of TLR4 expression and activation of its downstream signaling pathway [69]. These data are in agreement with our results, where leptin upregulation correlated with higher levels of TLR4 and MCP-1 expression, and increased TLR4 mRNA following leptin treatment (Fig 5). These results confirm the role of leptin in the upregulation of TLR4. Additionally, hepatocytes treated with ethanol and leptin had higher levels of MCP-1 mRNA, indicating the role of leptin in the up-regulation of MCP-1 in this model. In the literature, leptin has been shown to increase MCP-1 secretion from adipose stem cells in a dose- and time-dependent manner [70]. Furthermore, previous studies have indicated the role of TLR4 in the up-regulation of MCP-1. TLR4^-/-^ mice fed on high fat diet have been reported to have reduced hepatic macrophage infiltration with decreased MCP-1 and CCR2 expression [71]. Based on these results, it is likely that leptin plays an important role in the up-regulation of TLR4 and MCP-1 in our model.

**Fig 6:**
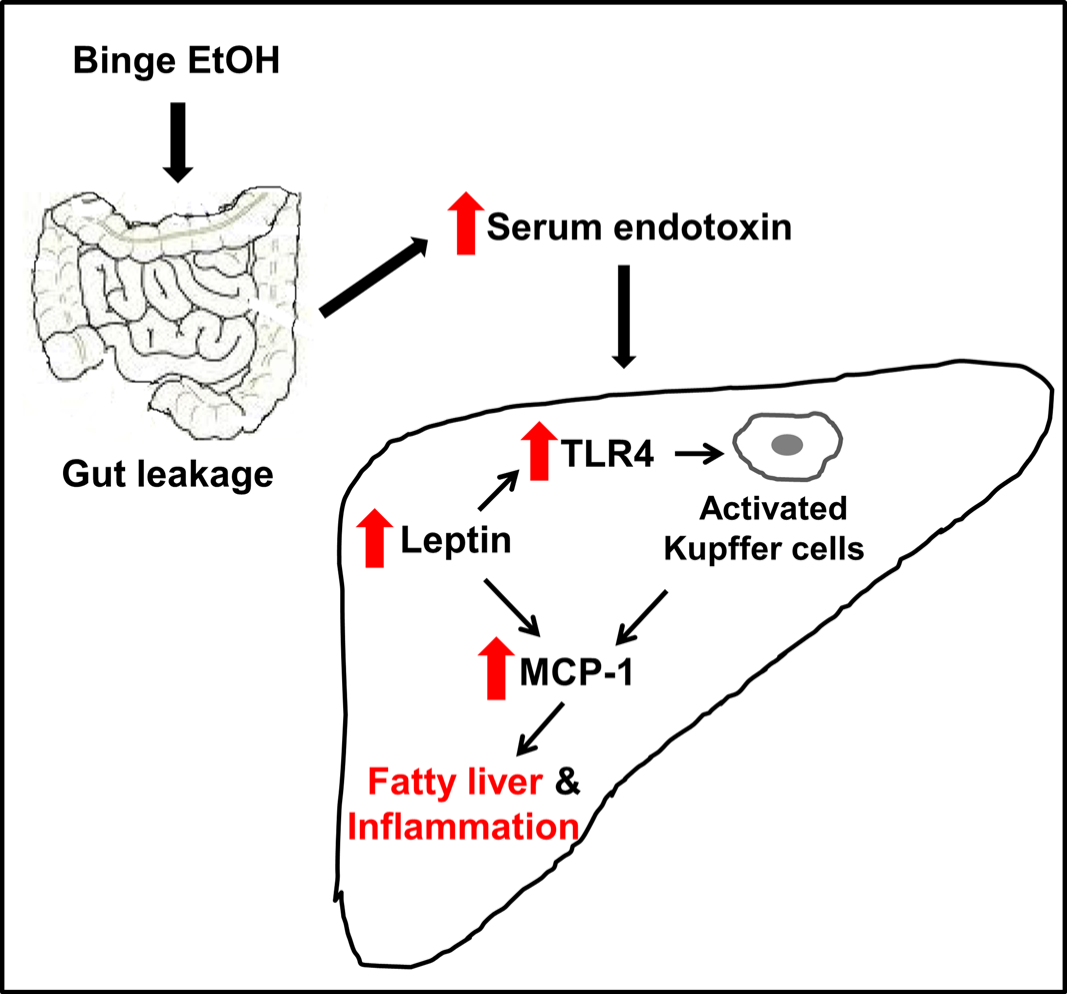
Schematic diagram describing a potential mechanism for binge alcohol-mediated gut leakiness, inflammation and steatosis in HIV-Tg rats. Binge alcohol can increase gut leakiness, serum endotoxin levels, the expression of TLR4 receptor, contributing to activation of Kupffer cells, producing greater amounts of MCP-1 and CCR2 for neutrophil infiltration and subsequently inflammatory fatty liver injury in HIV-Tg rats compared to the corresponding WT.

Taken together, our results showed that HIV-Tg rats are more susceptible to binge alcohol-induced gut leakiness accompanied with elevated endotoxin and steatohepatitic lesions compared to their WT counterparts, possibly due to additive or synergistic interaction between binge alcohol exposure and HIV infection. In the HIV-Tg rats, binge alcohol increases LPS/endotoxin-mediated hepatic leptin and TLR4 level, which further activated Kupffer cells with up-regulated levels of MCP-1 and CCR2, at least partially, contributing to greater hepatic inflammation and steatosis. Collectively, these results provide the first evidence for one of the mechanisms of increased gut leakage and inflammatory hepatic injury in HIV-Tg rats exposed to binge alcohol (Fig 6), similar to the pathophysiological states of HIV-infected individuals on HAART. Based on these results, HIV-Tg rats can be used as a surrogate model to study the underlying mechanisms of many pathophysiological conditions caused by heavy alcohol intake in HIV-infected people on HAART.

## Supporting Information

S1 Fig Serum levels of major cytokines in the control and ethanol-treated WT and HIV-Tg rats. Age-matched WT and HIV-Tg rats were exposed to 3 doses of dextrose (control) or ethanol (3.5 g/kg oral gavage at 12-h intervals, n≥4/group) and blood from each rat was collected at 1 h after the last dose of treatment. The serum level of the indicated cytokine/chemokine in each rat was determined by the method as described in the Materials and Methods. *, # Significantly different from the corresponding dextrose controls and ethanol-exposed WT counterparts, respectively.
(DOC)
