# Supplementary material for: Increased Sensitivity to Binge Alcohol-Induced Gut Leakiness and Inflammatory Liver Disease in HIV Transgenic Rats
Source: PLoS One. 2015 Oct 20;10(10):e0140498. doi: 10.1371/journal.pone.0140498 (PMC4618849; doi:10.1371/journal.pone.0140498)
Supplement: S1 Fig — Age-matched WT and HIV-Tg rats were exposed to 3 doses of dextrose (control) or ethanol (3.5 g/kg oral gavage at 12-h intervals, n≥4/group) and blood from each rat was collected at 1 h after the last dose of treatment. The serum level of the indicated cytokine/chemokine in each rat was determined by the method as described in the Materials and Methods. *, # Significantly different from the corresponding dextrose controls and ethanol-exposed WT counterparts, respectively. (DOC) [file pone.0140498.s001.doc]

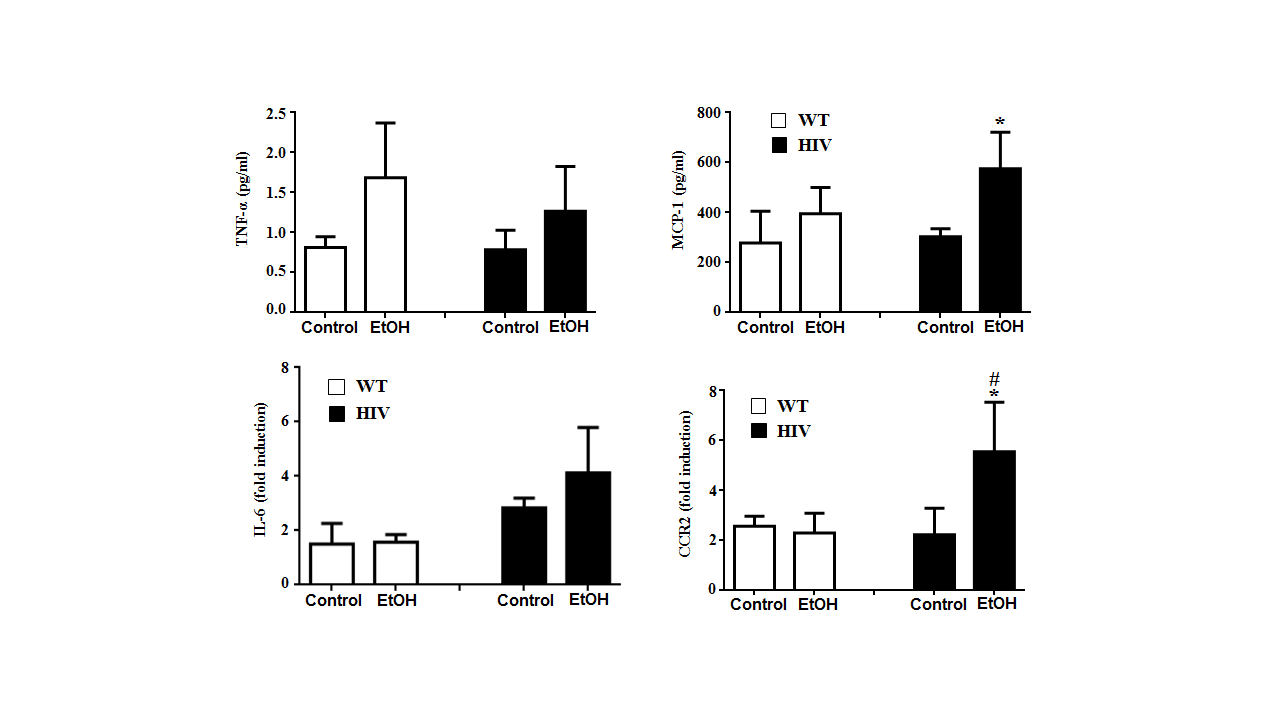


**S1 Fig. Serum levels of major cytokines in the control and ethanol-treated WT and HIV-Tg rats**. Age-matched female WT and HIV-Tg rats were treated with 3 doses of dextrose (control) or ethanol (3.5 g/kg oral gavage at 12-h intervals, n≥4/group) and blood from each rat was collected at 1 h after the last dose of treatment. The serum level of the indicated cytokine/chemokine in each rat was determined by using the Multiplex bead array assay kit, as described in the Materials and Methods. The results represent means ± SEM for the indicated groups. *, # Significantly different from the corresponding dextrose controls and ethanol-exposed WT counterparts, respectively.
